# Integrated Flow Enzymatic Immobilization and Esterification for Synthesizing 5‑Hydroxymethylfurfural Oleate

**DOI:** 10.1021/acsomega.6c09089

**Published:** 2026-09-22

**Authors:** Karina Reichel, Nadia Guajardo, Carolina Aguirre

**Affiliations:** † Departamento de Ingeniería Química y Bioprocesos, Escuela de Ingeniería, Pontificia Universidad Católica de Chile, Avenida Vicuña Mackenna 4860, Macul, Santiago 7820436, Chile; ‡ Centro de Energía, Departamento de Química Ambiental, Facultad de Ciencias, 28045 Universidad Católica de la Santísima Concepción, Concepción 4090541, Chile

## Abstract

Enzyme immobilization can be carried out in either batch or flow mode. Developing flow-through enzymatic reactions requires integrating upstream flow bioprocesses, such as flow immobilization, to minimize downtime, reduce labor requirements, and support the development of sustainable bioprocesses. In this study, *Burkholderia cepacia* lipase (BCL) was immobilized on Immobead 150 P, and flow esterification of 5-hydroxymethylfurfural (HMF) with oleic acid was performed to synthesize 5-hydroxymethylfurfural oleate. For batch immobilization, the optimal enzyme loading was determined to be 7 mg protein per gram of support, yielding an activity yield (IY%) of 99.9% and a protein yield (IP%) of 98.6%. In flow mode, the best immobilization results were achieved at a flow rate of 0.05 mL/min, yielding an IY of 98% and an IP of 80% within 130 min. The resulting biocatalyst exhibited high operational stability, maintaining activity after five reaction cycles. Batch synthesis of 5-hydroxymethylfurfural oleate achieved conversions of 97% and 45% using 30 mM and 50 mM HMF, respectively, over 24 h. In flow esterification of HMF and oleic acid, conversions of 84% (30 mM HMF) and 75% (50 mM HMF) were obtained at a flow rate of 0.05 mL/min. The space-time yield (STY) was higher in flow bioreactors, with values of 4.7 mM/min and 5.3 mM/min at the same flow rate for 30 mM and 50 mM HMF, respectively. This work demonstrates the integration of two flow processes—lipase immobilization and HMF esterification with oleic acid—highlighting the significant potential for process scale-up.

## Graphical abstract

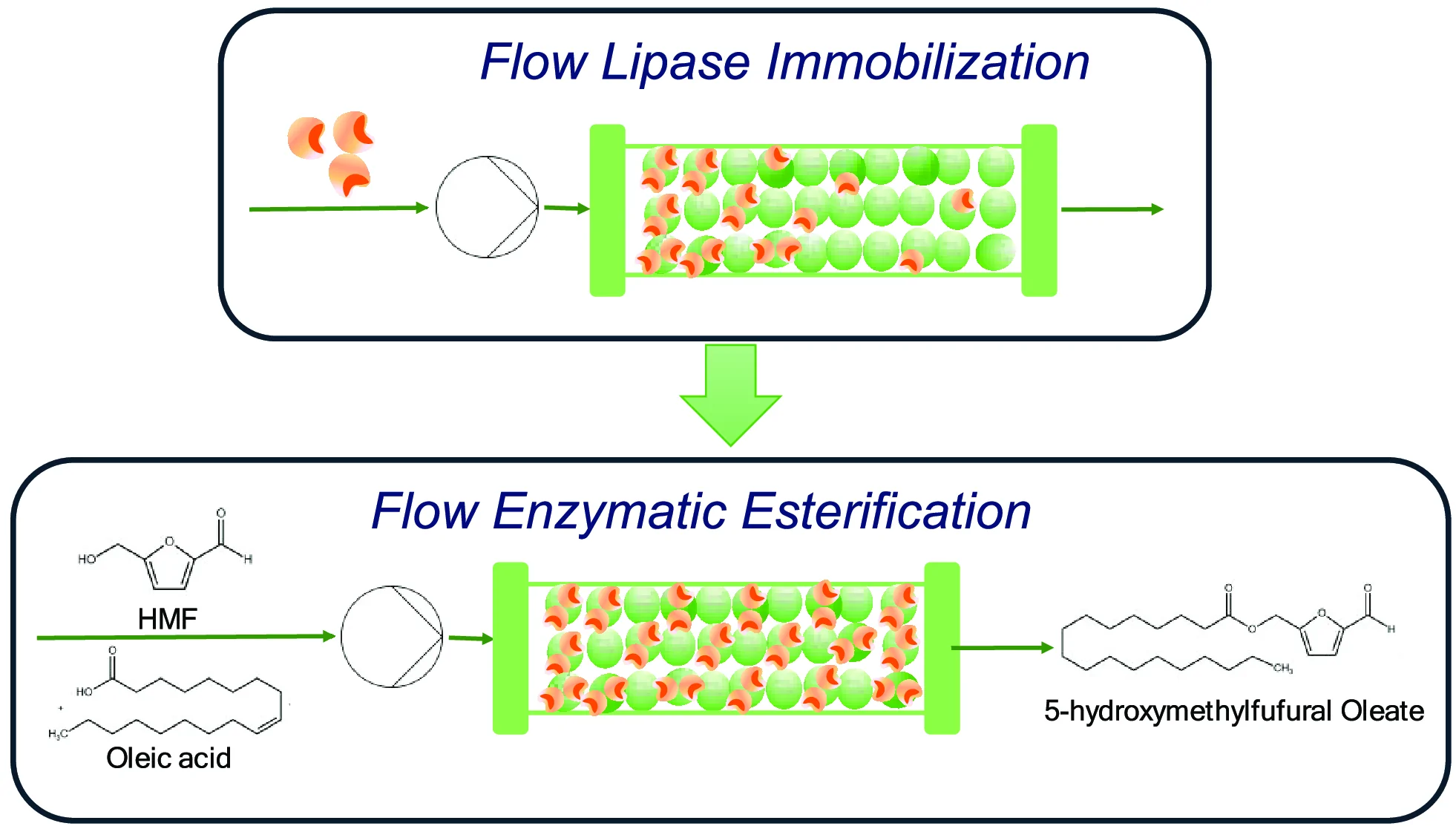

## Introduction

The adoption of biocatalysis is expanding across diverse industries due to rising demand for sustainable and highly efficient processes that reduce waste.^1,2^ However, its industrial application in production lines requires strategies to ensure stabilization, productivity, and profitability.^3^ One such stabilization strategy is enzyme immobilization, which also enables biocatalyst reuse and the operation of various bioreactor types.^4^ The traditional method for immobilization is batch mode.^4,5^ However, in flow systems, batch immobilization is not the most suitable approach, as it can negatively impact process productivity.^6–8^ Furthermore, flow immobilization minimizes downtime, decreases labor requirements, streamlines control systems, and facilitates the advancement of sustainable bioprocesses. In this context, packed-bed enzymatic bioreactors are ideal for flow immobilization, as they allow for online operation.^9^ Other advantages of flow bioprocesses include ease of scale-up and the ability to integrate downstream processes, producing a product with consistent characteristics.^10–13^

Among the immobilization methodologies, physical and chemical bonding stand out.^6,14–18^ Physical methods involve weak van der Waals forces and hydrophobic interactions,^19,20^ while chemical methods rely on the formation of covalent bonds between the enzyme and the support.^21–23^ Of these two approaches, chemical methods provide greater stability, as the enzyme cannot detach from the support. The range of supports for enzyme immobilization is diverse, encompassing polymers, biopolymers, and, increasingly, sustainability-focused materials derived from agro-industrial waste.^24–26^

Selecting the appropriate support for enzyme immobilization depends on several factors, including the type of bioreactor to be used, the specific reaction to be catalyzed, and the stability and activity of the biocatalyst.^4,5^ For example, in continuous stirred-tank bioreactors, the operational stability of the support is crucial to prevent biocatalyst degradation.^27–29^ In packed-bed bioreactors, factors such as particle size, mechanical strength, and support polarity can influence the retention of certain reaction products.^30,31^ In fluidized-bed bioreactors, the biocatalyst must remain properly suspended within the catalytic bed, which is why magnetic supports are often employed.^11^ In this study, Immobead 150 P was chosen because it enables covalent immobilization of the lipase, offers a suitable particle size for flow operation, and possesses good mechanical strength.^32,33^

The declining availability of fossil-based resources for energy production and the synthesis of commercially valuable compounds has accelerated the search for sustainable alternatives that utilize renewable feedstocks.^34–37^ One promising avenue is the conversion of lignocellulosic waste, generated during bioethanol production, into valuable furans.^38^ Among these, 5-hydroxymethylfurfural (HMF) stands out as a key platform chemical. HMF can be further transformed into a variety of high-value products, including bioplastics, liquid fuels, and esters, highlighting its potential to support a circular, biobased economy.^39–43^

There are chemical and biological methodologies for transforming HMF. Chemical esterification involving HMF is challenging due to its high reactivity, which often leads to side reactions and byproduct formation.^44^ In this context, biocatalysis stands out as a concrete alternative for achieving selective and sustainable reactions.^45–47^ Lipase-catalyzed esterification is an environmentally friendly strategy for transforming HMF into 5-Hydroxymethylfurfural oleate.^45,47^ Applications of 5-Hydroxymethylfurfural oleate include biolubricants, biofuel blends, and fungicides.^29,45,47^ Motivated by the possibility of having new biobased products, the main objective of this work is to integrate the immobilization and enzymatic esterification of HMF and oleic acid in flow mode (Figure 1). While esterifications of HMF with other fatty acids have been reported,^46^ reactions with oleic acid have not been explored, nor have they been catalyzed by continuously immobilized *Burkholderia cepacia* lipase (BCL). Oleic acid is chosen over other fatty acids because it is a liquid fatty acid at room temperature, which gives the product different characteristics.

**Figure 1:**
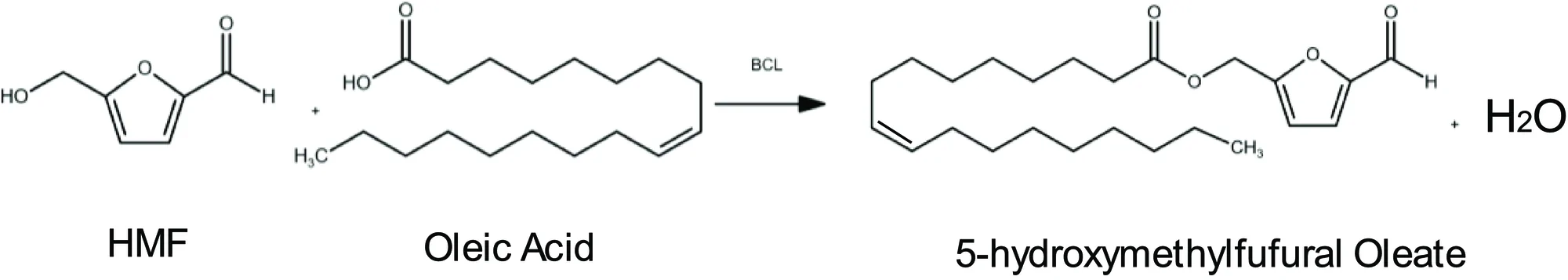
Enzymatic synthesis of 5-hydroxymethylfurfural oleate catalyzed by *Burkholderia cepacia* *lipase*.

## Material and Methods

### Chemicals

HMF, oleic acid (90%), Immobead 150 P, and *Burkholderia cepacia* lipase were purchased from Sigma–Aldrich and used without further modification. Formic acid and HPLC-grade methanol were obtained from Merck S.A., Santiago, Chile.

### Analytical Method for the Quantification of HMF

The reaction products were analyzed by HPLC using a JASCO LC-4000 system equipped with a diode array detector and a GL Sciences C_18_ HPLC column (100 × 4.6 mm I.D.) with detection at 285 nm. Separation was achieved using a mobile phase composed of solutions A and B at an 85:15 (A: B) ratio, where A was water/formic acid (99.5/0.5), and B was methanol (100). Samples were diluted with the mobile phase, filtered with 0.22 μm membranes, and then loaded into the HPLC autosampler for analysis. The analyses were performed at a flow rate of 0.6 mL/min and a column temperature of 35 °C, resulting in a retention time of approximately 5 min for HMF (further details in Supporting Information 2).^29,45,46^

### Lipase Activity Assay

Enzyme activity was assessed spectrophotometrically using *p*-nitrophenyl butyrate (*p*-NPB) as the substrate. The hydrolysis of *p*-NPB (0.3 mM) in a 0.1 M phosphate buffer (pH 7) at 30 °C produces *p*-nitrophenol (*p*-NP), which is detected at 348 nm with a molar extinction coefficient (ε) of 5150 (M^–1^ cm^– 1^) using a Jasco V-730 spectrophotometer. One unit (*U*) of lipase activity is defined as the amount of enzyme required to hydrolyze one μmol of *p-*NPB per minute. The activities of free and immobilized lipase are determined using eqs 1 and 2, respectively.

1 $$\mathrm{Fr}ee\;enzyme\;activity\;\left(\frac{U}{L}\right)=\frac{\Delta A\times d_{f}\times V_{t}}{\varepsilon \times t\times L\times V_{s}}$$

2 $$Immobilized\;enzyme\;activity\;\left(\frac{U}{g}\right)=\frac{\Delta A\times d_{f}\times V_{t}}{\varepsilon \times t\times L\times m}$$

Where Δ*A* = change in absorbance, *d_f_* = dilution factor, *V_t_* = reaction volume (*L*), *V_s_* = sample volume (*L*), ε = molar extinction coefficient (M ^– 1^ cm^– 1^), *t* = reaction time (min), *L* is the optical path (cm), and *m* = amount of immobilized enzyme (*g*).

### Lipase Immobilization in Batch Mode

Lipase immobilization on Immobead 150 P support in batch mode was performed as follows: 1 g of support was mixed with 10 mL of enzyme solution (0.15 mg _Protein_/mL) in 100 mM phosphate buffer at pH 7, with gentle stirring in a roller shaker for 24 h at room temperature. The resulting biocatalyst was then washed three times with citrate buffer and stored at 4 °C. The activity yield is defined as the percentage of the immobilized enzyme’s activity relative to the free enzyme’s initial activity before immobilization. The protein yield is defined as the percentage of protein present in the immobilized enzyme relative to the free enzyme’s initial protein concentration before immobilization. The experimental tests were performed in triplicate, with the corresponding standard deviations (RSD). Activity and protein yields are calculated using eqs 3 and 4, respectively.

3 $$IY\;\left(\%\right)=\left[\frac{{(A}_{F}-A_{R})}{A_{F}}\right]\times 100$$

4 $$IP\;\left(\%\right)=\left[\frac{\left(P_{F}-P_{R}\right)}{P_{F}}\right]\times 100$$

Where IY(%) is the activity yield, IP(%) is the protein yield, *A*_F_ is the activity of the initial free enzyme, and *A*_R_ is the residual activity of the free enzyme. *P*_F_ is the initial protein concentration, and *P*_R_ is the residual protein concentration.

### Lipase Immobilization in Flow Mode

**L**ipase immobilization on Immobead 150 P support in flow mode was performed as follows: 1 g of support was mixed with 10 mL of enzyme solution (0.15 mg _Protein_/mL) in 100 mM phosphate buffer at pH 7. This mixture was introduced into a stainless-steel column (0.5 cm internal diameter, 14.4 cm length, volume of 2.86 cm^3^), packed with Immobead 150 P support at room temperature, using a peristaltic pump at a flow rate of 0.02–0.1 mL/min. Once the entire enzyme solution had passed through the column, the column—now containing the support and immobilized enzyme—was washed with 100 mM phosphate buffer, pH 7. The activity immobilization yield (IY, %) and protein immobilized yield (IP, %) were determined by collecting samples at different times and using eqs 3 and 4, respectively.

### Synthesis of 5-Hydroxymethylfurfural Oleate in a Batch Bioreactor

The enzymatic esterification of HMF with oleic acid was performed in batch mode (Figure 2) using a sealed reactor placed on a shaker at 165 rpm and 40 °C. The reaction medium consisted of 700 mg of biocatalyst (prepared by batch immobilization) and 10 mL of an oleic acid (90%) solution containing HMF (50 mM). The reaction was initiated by adding the biocatalyst, and samples were periodically withdrawn for product analysis.

**Figure 2:**
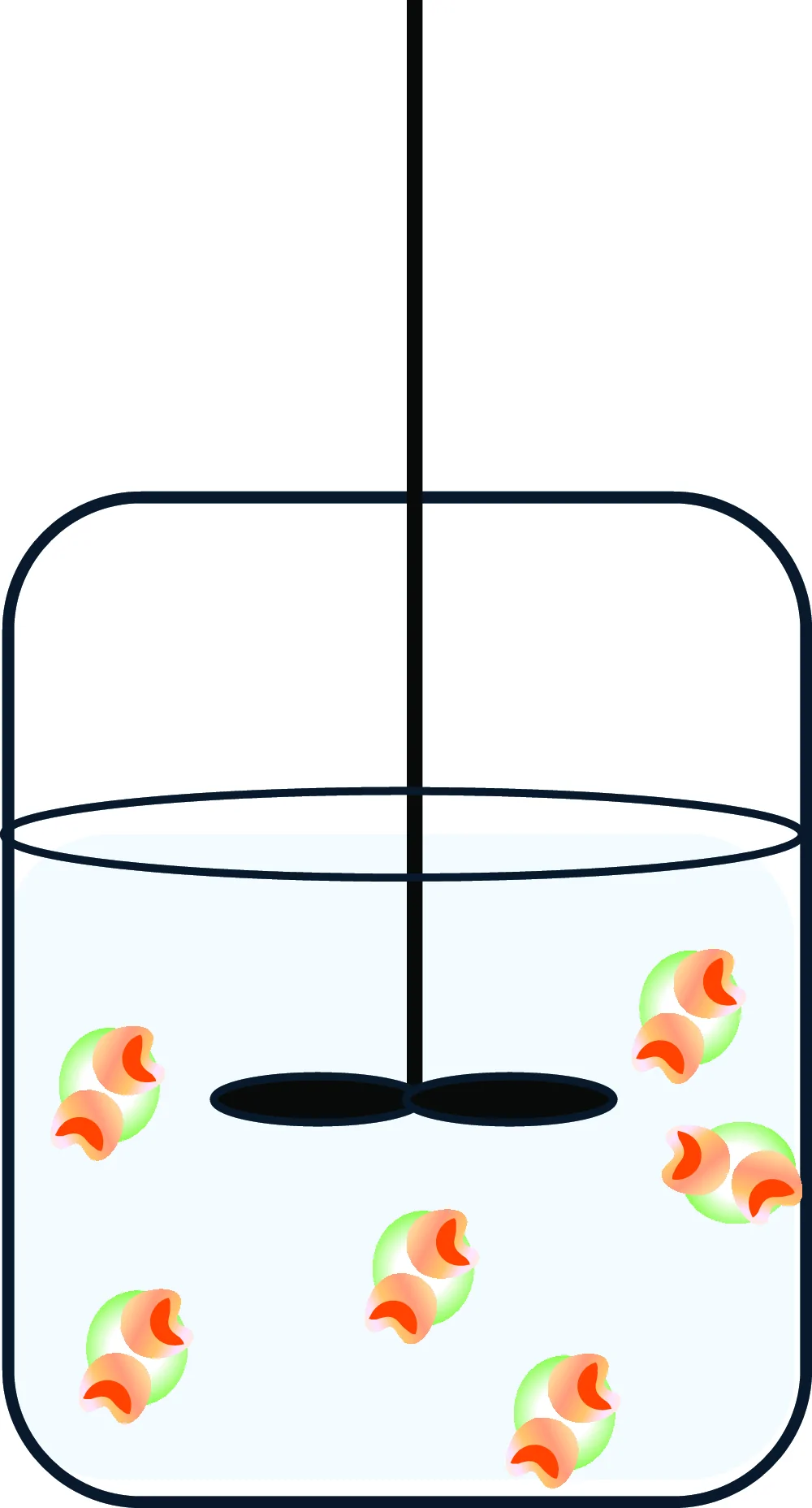
Enzymatic esterification of HMF with oleic acid catalyzed by the BLC enzyme immobilized in a stirred tank batch reactor.

The conversion and productivity were calculated using the eqs 5 and 6, respectively

5 $$\mathrm{Conversion}\;\left(\%\right)=\left[\frac{n_{\mathrm{so}}-n_{s}}{n_{s0}}\right]\times 100$$

where *n*_*S*0_ is the initial amount of substrate (HMF) and where *n*_*S*_ is the amount of substrate *S* at a given reaction time.

6 $$\mathrm{Productivity}=\frac{M}{t\cdot m_{\mathrm{biocatalyst}}}$$

Here, *M* is the mass of the product formed, *m*_biocatalyst_ is the mass of the biocatalyst, and *t* is the reaction time.

### Operational Stability of Immobilized BCL

The operational stability of the immobilized BCL was evaluated in batch mode. The reaction medium consisted of 3 mL of oleic acid (90%) containing 30 mM HMF, and 500 mg of biocatalyst was used at 40 °C for 24 h. After each reaction, the biocatalyst was recovered by filtration, washed with oleic acid, and used for five consecutive cycles with fresh reaction medium. To maintain consistent reaction conditions across batches, the ratio of biocatalyst (mg) to reaction medium volume (mL) was adjusted as needed. The reaction conversion was determined using eq 5.

### Synthesis of 5-Hydroxymethylfurfural Oleate in a Flow-Packed Bed Bioreactor

The enzymatic flow esterification of HMF with oleic acid to obtain 5-hydroxymethylfurfural oleate was performed in a stainless-steel column (0.5 cm internal diameter and 14.4 cm long) that was manually packed with the biocatalyst, as illustrated in Figure 3 (real-time picture in Figure S1). Similar to the batch bioreactor, the reaction medium was prepared by mixing HMF (30–50 mM) with 10–30 mL of oleic acid (90%) (volume of the reaction medium). The reactions were conducted at various flow rates (0.02–0.5 mL/min, controlled by a peristaltic pump), and samples were collected at defined time intervals. The column temperature was maintained in a column oven.

**Figure 3:**
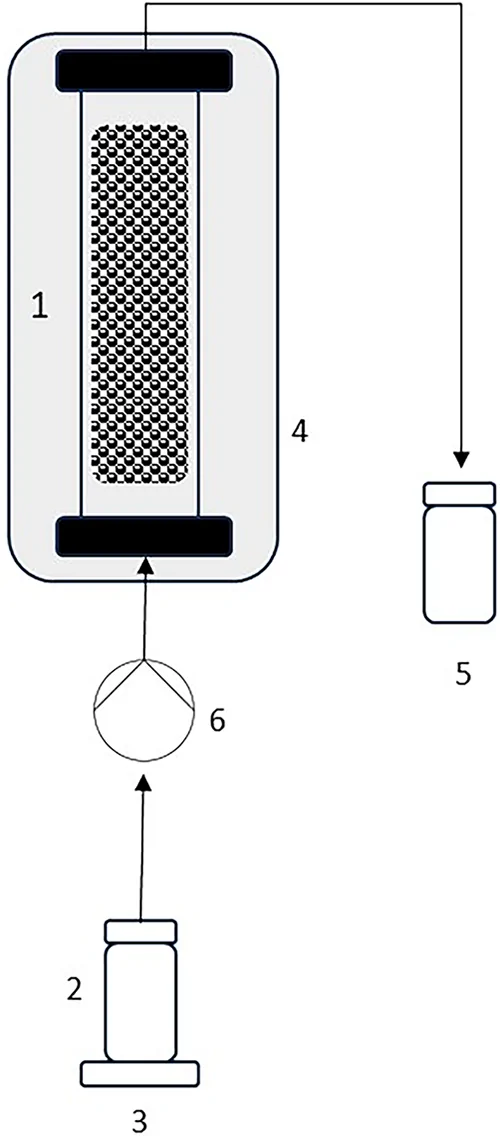
Flow-packed bed bioreactor set up. 1-Packed bed bioreactor, 2-Substrate feeding, 3- Heating plate, 4- Column oven, 5- Reaction products, 6-Peristaltic pump.

The residence times were determined via eq ([Disp-formula eq7])

7 $$\tau =\frac{\epsilon V_{t}}{q}$$

Where ϵ denotes the void fraction, *V*_*t*_ is the total bed volume, and *q* is the substrate flow rate. The void fraction of the packed-bed bioreactor used in this work was calculated using the average biocatalyst particle diameter, determined by scanning electron microscopy, and the correlation proposed by Benyahia and O’Neill.^48^ The conversion rate and productivity were calculated according to eqs 5 and 6.

The space-time yield of component *i* (STY_*i*_) in the flow-packed bed bioreactor, based on the liquid volume, is defined as eq 8.

8 $${\mathrm{ST}Y}_{i}=\frac{C_{P}}{\tau }$$

Where *C*_P_ is the product concentration.

## Results and Discussion

### Lipase Immobilization in Batch Mode

Table 1 presents the results of BCL immobilization in batch mode. Immobilization was performed using enzyme loads of 18.6 mg_Protein_/g_Support_, 7 mg_Protein_/g_Support_, and 1.5 mg_Protein_/g_Support_. The enzyme load that produced the highest activity and yield was 7 mg; therefore, this condition was chosen for flow immobilization. Generally, high enzyme loading can reduce biocatalyst activity, as saturation of the support with protein molecules may cause steric hindrance and mutual blocking of enzyme active sites. This observation could explain the results shown in Table 1. The amount of enzyme loaded onto a support also depends on the degree of enzyme purification^49^ and the nature of the support. One factor affecting enzyme activity after immobilization is that, at high protein concentrations, dimers or aggregates may form between enzyme molecules, obstructing the active site and decreasing biocatalyst activity.

**Table 1:** Batch Immobilization Parameters of BCL on Immobead 150P Support

Enzyme load (mg_Protein/_g_support_)	IY (%)	IP (%)	Activity (U/g_biocat._)	RSD (%)
**18.6**	99	98	2.06	1.9
**7**	99.9	98.6	7.3	1.5
**1.5**	87	98	5	2.1

The literature indicates that lipases immobilized on Immobead 150P generally achieve better results than those immobilized on other supports, although the effect of high enzyme loading can vary. For instance, Madalozzo et al.^50^ reported that lipase (LipC12) immobilized on Immobead 150P achieved a 15% higher conversion of biodiesel esters than the same enzyme immobilized on Accurel MP-1000, highlighting the effectiveness of the support. The same study found that a higher enzyme load produced better results.^50^ Conversely, other research, such as that by Sabi et al.,^51^ showed that Eversa Transform lipase immobilized on octyl agarose displayed greater specific activity at a lower protein load (1 mg/g support), demonstrating that optimizing the enzyme load can improve activity by up to 3-fold.^51^

### Lipase Immobilization in Flow Mode

The results of flow immobilization are presented in Figure 4. Various flow rates (0.1–0.02 mL/min) were tested, with the best results observed at 0.05 mL/min, yielding 98% activity and 80% protein yield after 105 min.

**Figure 4:**
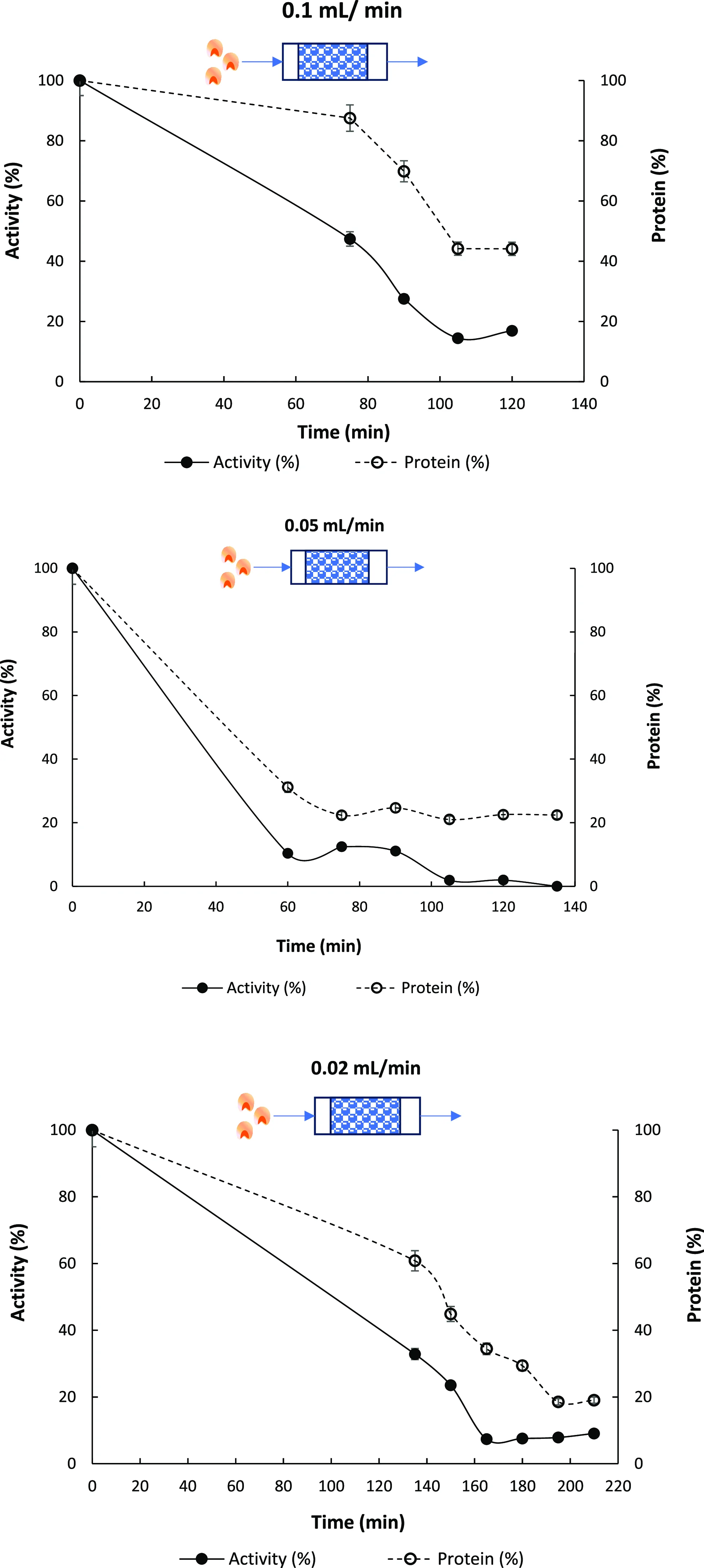
Flow immobilization kinetics of BCL lipase at different flow rates at room temperature.

The immobilization process achieved a higher activity yield at a flow rate of 0.05 mL/min and was completed in 105 min, the same duration observed for lipase immobilization at 0.1 mL/min. In contrast, at a flow rate of 0.02 mL/min, the process required approximately 150 min, which negatively impacted overall productivity.

There are few publications regarding flow immobilization. For example, Bussamara et al.,^20^ optimized the immobilization of a lipase on a polystyrene support in a packed-bed column. According to a response surface analysis, the optimal immobilization conditions were an enzyme-to-support ratio of 1282 U/g, a time of 150 min, and a flow rate of 2 mL/min.

Another example is the work of Freitas et al.,^52^ which reported the immobilization of an extracellular lipase in a packed-bed bioreactor. In this study, the optimal immobilization flow rate in the packed-bed bioreactor (0.5 cm internal diameter and 14.4 cm long, 1 g of support) was 1 mL/min. This flow rate was used to feed the bed with 125 mL of enzyme extract (3 mg protein/mL) for 24 h at room temperature (20 °C), with recirculation.

When comparing the optimal feeding rates obtained in our study with those reported in the literature, we found that our flow rates were significantly lower. However, these lower flow rates enabled us to achieve higher IY% and IP% without the need for recirculation. In contrast, studies in literature required enzyme recirculation to reach higher IY% and IP%. Another factor that may have influenced the process when working with nonpurified extracts is the possible immobilization of other proteins or contaminants besides lipase, which could decrease the biocatalyst’s activity.

### Synthesis of 5-Hydroxymethylfurfural Oleate in a Batch Bioreactor

Figure 5 illustrates the enzymatic esterification behavior in a batch reactor. At a HMF substrate concentration of 30 mM, enzymatic esterification achieves 84% conversion in 30 min; however, due to the low substrate concentration, it takes over 23 h to reach 96% conversion. A similar trend, but with a lower conversion (32%), was observed at 50 mM HMF. The high reaction rate is related to both the biocatalyst’s activity and the substrate concentration. At higher substrate concentrations, the enzyme degrades HMF more slowly in the presence of oleic acid, likely due to diffusional limitations. This phenomenon has also been reported in the literature. For instance, in the esterification of oleic acid with ethanol catalyzed by Novozym 435 lipase, a high enzyme load was shown to increase the initial reaction rate.^53^ Similarly, in the esterification of oleic acid with *n*-decanol catalyzed by immobilized *Candida rugosa* lipase, higher conversion rates were observed at the start of the reaction. The authors attributed this to optimal water activity, which enables the enzyme to maintain its open conformation and exposes the active site.^54^

**Figure 5:**
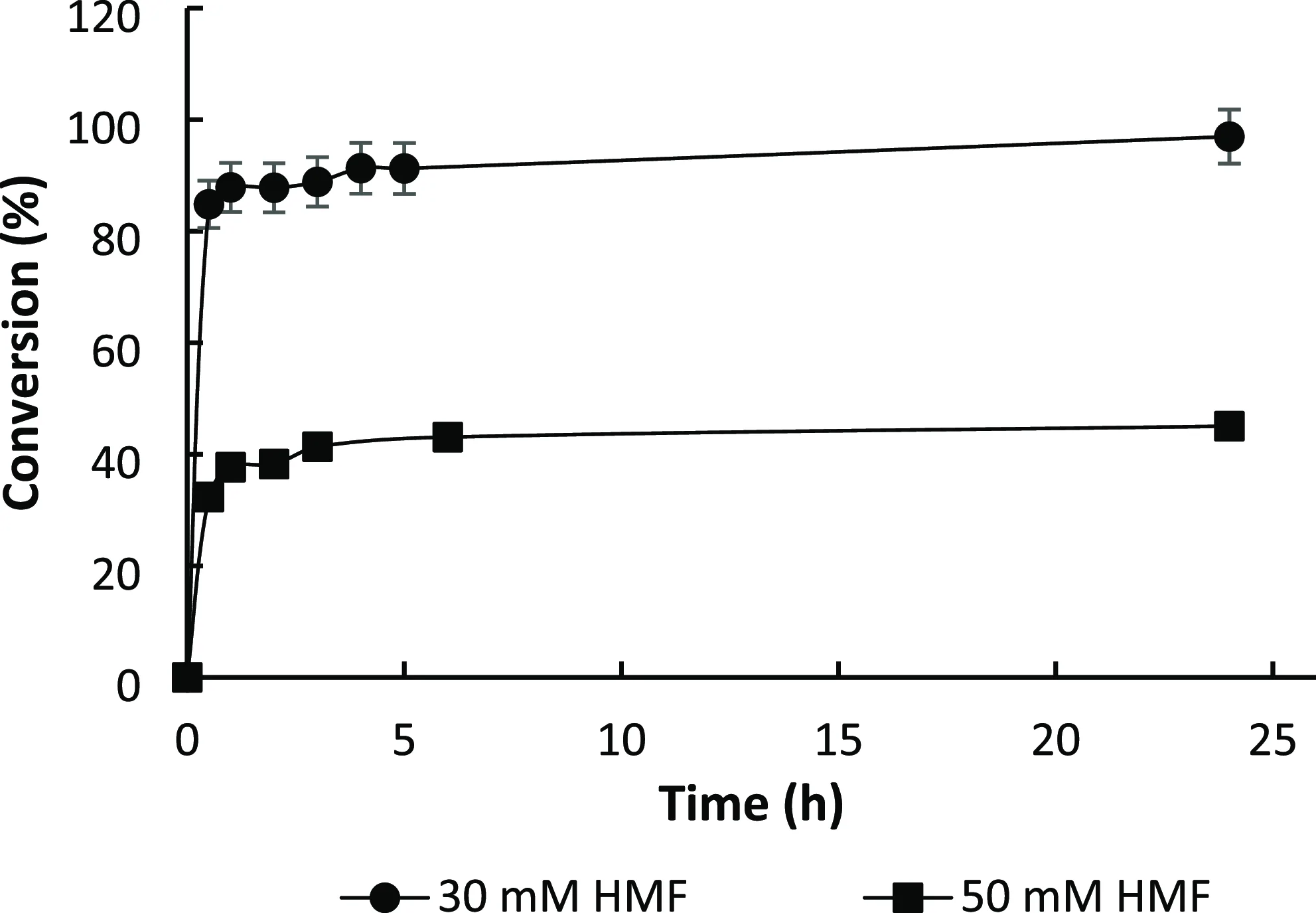
Synthesis of 5-hydroxymethylfurfural oleate in a batch bioreactor. Reaction conditions: 700 mg_Biocat._, 6 mL of oleic acid at 40 °C.

### Operational Stability of the Biocatalyst in a Batch Bioreactor

Maintaining biocatalyst activity during the reaction is crucial for process development and scalability. Therefore, it is necessary to evaluate the operational stability of the biocatalyst over multiple reaction cycles. Figure 6 shows that the biocatalyst remains active for 5 reaction cycles, enabling sustained activity throughout the reaction and supporting the development of flow processes. Similar results have been reported for other immobilized lipases. For example, Novozym 435, used to catalyze the esterification of propanol and oleic acid, retained 94% of its activity after five cycles.^55^ In the esterification of oleic acid with *n*-decanol catalyzed by *Candida rugosa* lipase immobilized on hemp tea waste, the biocatalyst maintained 60% of its activity after six reaction cycles, likely due to the immobilization method—adsorption—which does not involve covalent bonding to the support, allowing the enzyme to detach.^54^ These findings indicate that the immobilization methodology is a key factor for enzyme stability. In the case of BCL lipase immobilized on Immobead 150 P, its high stability may be attributed to multipoint covalent bonding between the enzyme and the support, which contains epoxide groups on its surface.

**Figure 6:**
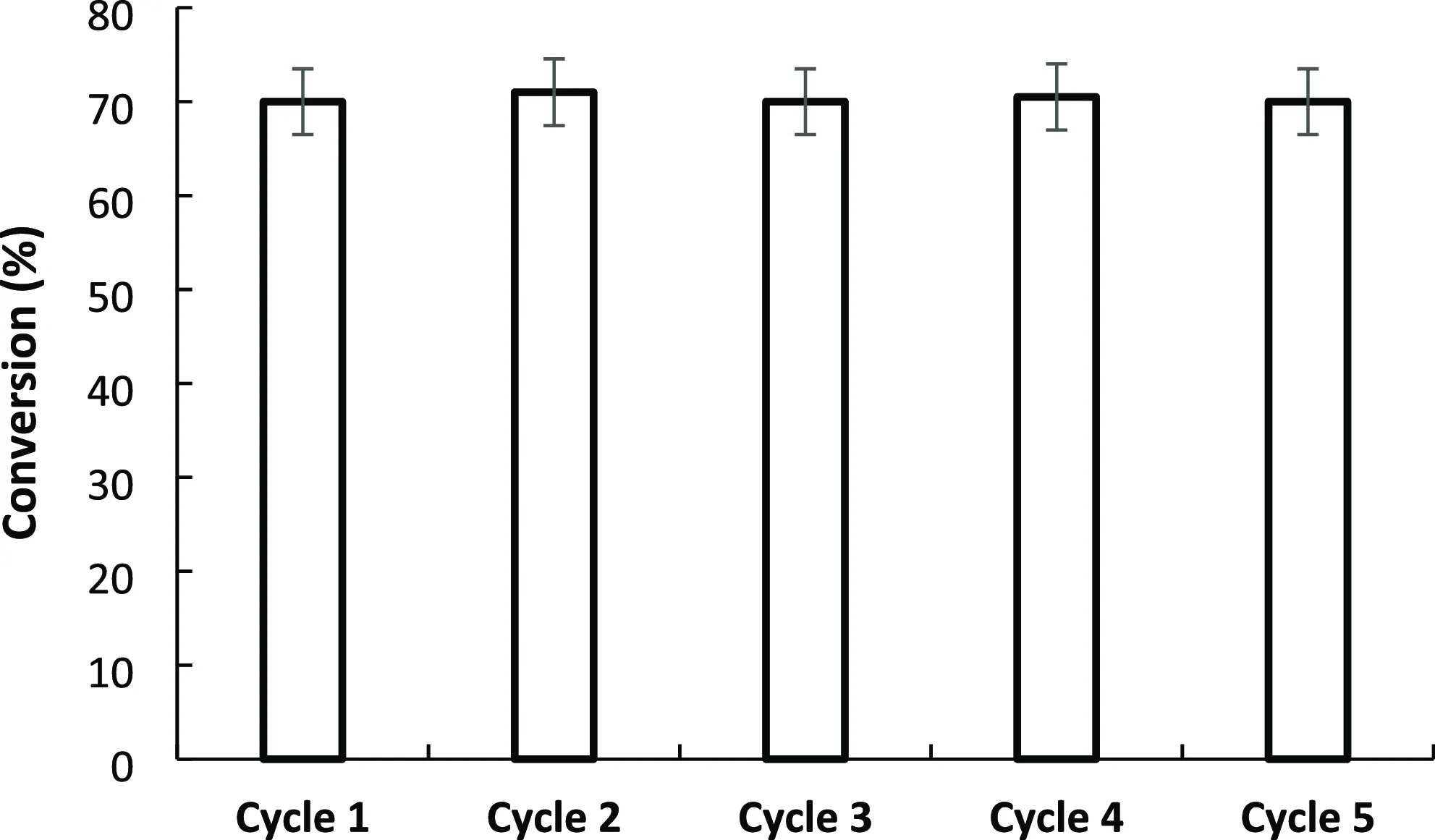
Operational stability of immobilized BCL in a batch bioreactor. Reaction conditions: 30 mM HMF, 500 mg Biocat., 3 mL of oleic acid, at 40 °C for 24 h.

### Synthesis of 5-Hydroxymethylfurfural Oleate in a Flow-Packed Bed Bioreactor

After confirming the biocatalyst’s stability, experimental tests were performed in the flow-packed bed bioreactor (Figure 6). In Figure 7A, the best results were obtained using a substrate concentration of 30 mM and flow rates of 0.02 mL/min and 0.05 mL/min, respectively. These results indicate that, within this range, the flow rate does not significantly affect reaction conversion, which is favorable for process productivity. However, increasing the flow rate to 0.1 mL/min results in a 10% decrease in conversion. When using an HMF concentration of 50 mM, the conversion rate decreased by 6%. This small difference suggests that, with further optimization, the system could achieve higher conversion rates and productivity, enhancing its industrial relevance. Figure 7B presents the reaction kinetics of the esterification of oleic acid and HMF in the packed-bed flow bioreactor over 25 h of operation. The results show no loss of enzymatic activity, as conversion remains constant throughout the process. It is also noteworthy that the difference in conversion observed between 30 mM and 50 mM HMF substrate concentrations is minimal at a flow rate of 0.05 mL/min.

**Figure 7:**
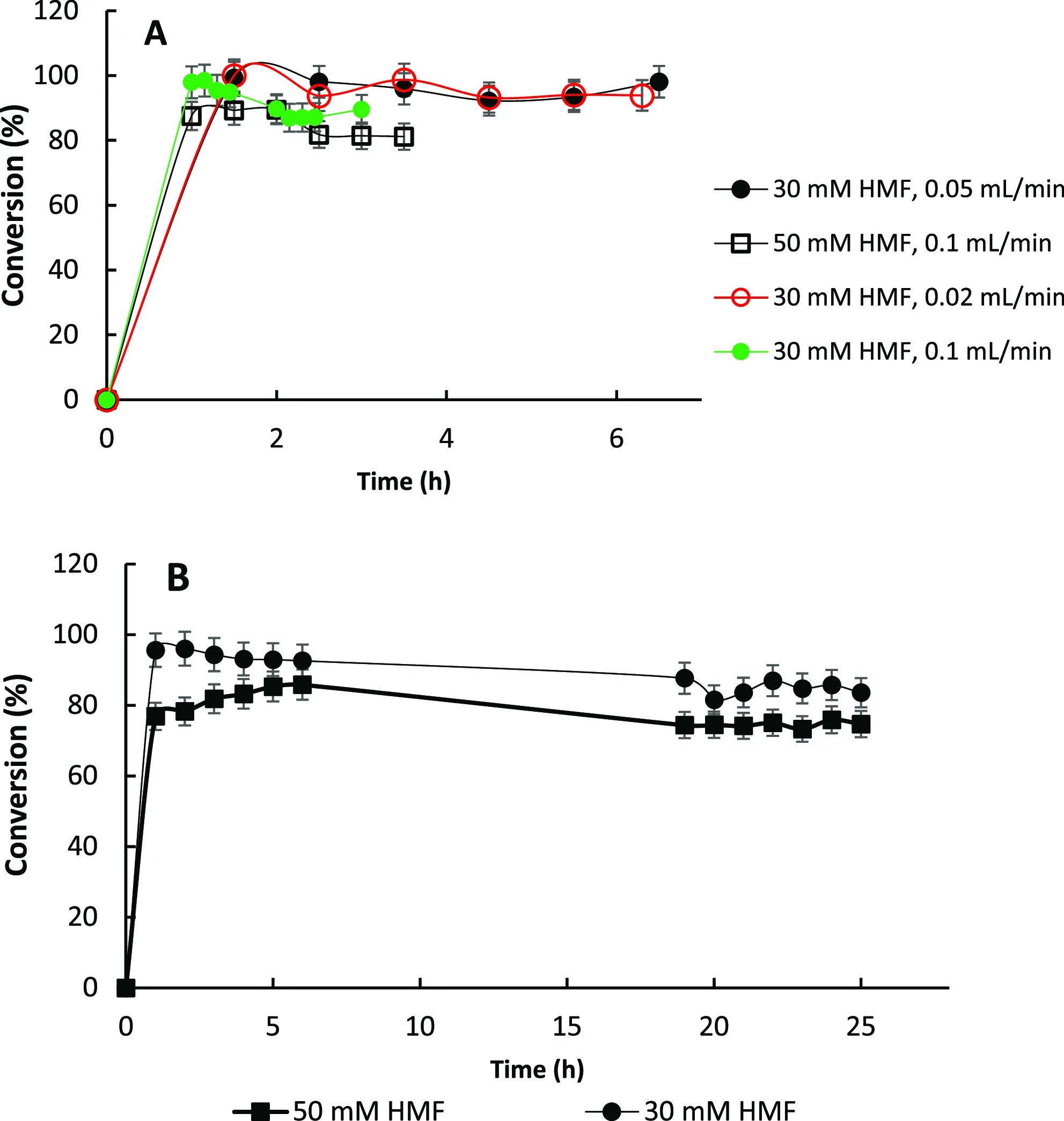
Synthesis of 5-hydroxymethylfurfural oleate in a flow-packed bed bioreactor. (A) at different HMF concentrations, 20 mL of oleic acid at 40 °C; (B) operational stability of BCL immobilized in a flow-packed bed bioreactor. Reaction conditions: 80 mL of oleic acid, 50 mM HMF at 0.05 mL/min.

The conversion achieved in the packed-bed flow bioreactor was even higher than those previously reported. For instance, in the enzymatic esterification of isoamyl alcohol with oleic acid catalyzed by Novozym 435 in a flow microreactor, a 45% conversion was maintained over 140 h of continuous operation.^56^ These findings on stability in flow mode are consistent with the results obtained in our study.

The esterification of oleic acid with glycerol to produce diacylglycerol in a packed-bed flow bioreactor catalyzed by the enzyme Novozym 435 achieved conversions of 85% at low flow rates (2 × 10^–5^ L/min). However, operational stability decreased by 6% after 11 days of operation, possibly due to biocatalyst inactivation.^57^ As observed in this research, these results demonstrate the high stability of immobilized lipases in catalyzing oleic acid esterifications, achieving both high conversions and operational stability.

A comparison of bioreactor performance in terms of STY (Figure 8) demonstrates that flow-packed-bed bioreactors yield the best results, allowing for intensified enzymatic esterification. The findings indicate a trade-off between conversion and STY*_i_*. For instance, in the esterification conducted in a packed-bed flow bioreactor, conversion was higher with 30 mM HMF than with 50 mM HMF. However, at the same flow rate, the STY*_i_* was greater with 50 mM HMF, resulting in a higher amount of 5-hydroxymethylfurfural oleate produced.

**Figure 8:**
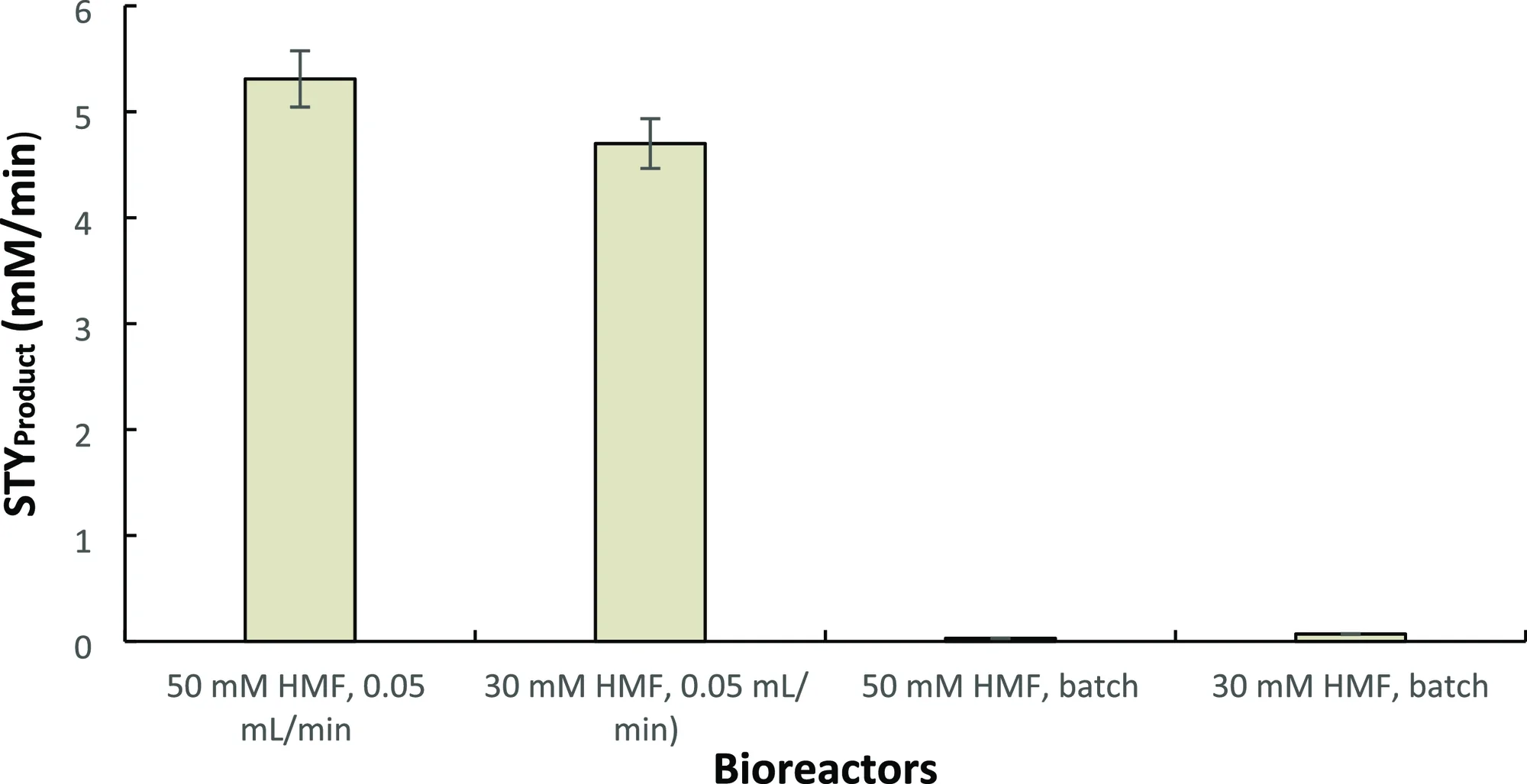
Space-time yield (STY) of 5-Hydroxymethylfurfural oleate with different types of bioreactors at 40 °C.

Flow operation increases the productivity of 5-hydroxymethylfurfural oleate, highlighting its significant potential for scale-up.

## Conclusions

The present study successfully demonstrated the feasibility of 5-Hydroxymethylfurfural Oleate catalyzed by immobilized lipase BCL in a flow system, integrating both immobilization and enzymatic esterification in flow mode. Under flow immobilization, at 0.05 mL/min, IY of 98% and IP of 80% were obtained, results comparable to those obtained in batch-mode immobilization. The synthesis of 5-hydroxymethylfurfural oleate in batch mode using 30 mM and 50 mM HMF resulted in conversions of 97% and 45%, respectively, within 24 h. BCL lipase immobilized on Immobead 150P also exhibited high stability over five reaction cycles. In flow esterification using a packed-bed bioreactor, conversions of 84% (30 mM HMF) and 75% (50 mM HMF) were achieved after 25 h. Additionally, the STY indicated that flow operation yields higher values than batch bioreactors, enabling intensified processing. This research contributes to the development of integrated immobilization and flow esterification processes with the potential for industrial-scale production of 5-hydroxymethylfurfural oleate.

## Supplementary Material



## Data Availability

The data that support this study are available from the corresponding author, Nadia Guajardo, upon reasonable request.

## Notes

Packed-bed flow bioreactor setup (Figure S1); chromatogram of the reaction mixture before starting the enzymatic esterification (Figure S2); chromatogram of the reaction mixture at the end of the enzymatic esterification (Figure S3); FT-IR spectrum before the reaction started (black line) and at the end of the reaction (red line) (Figure S4); 1H-NMR of the reaction samples: (A) before starting the reaction and (B) after the reaction was completed (Figure S5); 13C-NMR of the samples before (Figure S6A) and after the reaction (Figure S6B) (Figure S6) (DOCX)
