# Supplementary material for: Integrated Flow Enzymatic Immobilization and Esterification for Synthesizing 5‑Hydroxymethylfurfural Oleate
Source: ACS Omega. 2026 Sep 22;11(39):60438–45. doi: 10.1021/acsomega.6c09089 (PMC13647920; doi:10.1021/acsomega.6c09089)
Supplement: Supplementary file 1 [file ao6c09089_si_001.docx]

**Integrated Flow Enzymatic Immobilization and Esterification for Synthesizing 5-Hydroxymethylfurfural Oleate**

Karina Reichel^a^, Nadia Guajardo*^a^, and Carolina Aguirre^b^

^a:^ Departamento de Ingeniería Química y Bioprocesos, Escuela de Ingeniería, Pontificia Universidad Católica de Chile, Avenida Vicuña Mackenna 4860, Macul, Santiago, Chile.

^b:^ Centro de Energía, Departamento de Química Ambiental, Facultad de Ciencias, Universidad Católica de la Santísima Concepción, Concepción, Chile.

* Corresponding Author: Prof. Dr. Nadia Guajardo.

E-mail address: [nadia.guajardo@uc.cl](mailto:nadia.guajardo@uc.cl)

**Supplementary information**

1. **Continuous reaction system setup**

Figure S1 details the setup of the continuous reaction system.

**Figure S1.** Packed bed flow bioreactor setup

2. **Analysis of reaction products**

Figures S2 and S3 detail the chromatograms of the substrate (Figure S2) and the reaction product (Figure S3). As can be seen in Figure S2, the substrate HMF elutes at a retention time of 4.5 min, and the product 5-Hydroxymethylfurfural Oleate elutes at 21 min (Figure S3).

**Figure S2.** Chromatogram of the reaction mixture before starting the enzymatic esterification.

**Figure S3.** Chromatogram of the reaction mixture at the end of the enzymatic esterification

Figure S4 details the FT-IR spectra of the reaction samples before and after enzymatic esterification.

**Figure S4.** FT-IR spectrum before the reaction started (black line) and at the end of the reaction (red line)

Because the reaction mixture contains the substrates HMF, oleic acid, and 5-Hydroxymethylfurfural Oleate (reaction product), characterization is difficult. As shown in Figure S4, there is no distinct band between 1730 and 1750 cm⁻¹ (C=O) characteristic of the ester. Only the band at 1702 cm⁻¹, corresponding to the carbonyl groups (C=O) of oleic acid and also present in the reaction product, is clearly visible. What is observed is a difference in the intensity of the spectra, which may indicate the presence of 5-Hydroxymethylfurfural oleate.

Figure S5 details the 1H NMR spectra of the samples before the reaction (Figure S5A) and after the reaction was completed (Figure S5B). It was observed that the CH₂ α to the carbonyl remains at 2.29–2.33 ppm in both spectra, while the olefinic signal remains at 5.31 ppm. No new, clear signal is observed in the 4.0–5.2 ppm region attributable to a –CH₂–O–CO– group generated by esterification. Importantly, for an ester derived from oleic acid, the signal from the protons of the double bond remains at approximately 5.3 ppm in both the acid and the ester. Therefore, this signal demonstrates the presence of an unsaturated oleic acid chain, but it does not allow for differentiation from the esterified oleate.

**Figure S5.** 1H-NMR of the reaction samples. A) before starting the reaction and B) after the reaction was completed.

Figure S6 details the 13C-NMR of the samples before (Figure S6A) and after the reaction (Figure S6B). The same signals can be distinguished in both spectra: 14.1 ppm: terminal CH₃, 22–34 ppm: CH₂ carbons of the aliphatic chain, 127.9–130.5 ppm: carbons of the oleic acid double bond, 76.7–77.3 ppm: triplet of the solvent CDCl₃, not corresponding to the product. The same signals can be distinguished in both spectra: 14.1 ppm: terminal CH₃, 22–34 ppm: CH₂ carbons of the aliphatic chain, 127.9–130.5 ppm: carbons of the oleic acid double bond, 76.7–77.3 ppm: triplet of the solvent CDCl₃, not corresponding to the product. In the final spectrum (Figure S6B), some additional or better-resolved aliphatic signals appear, for example, around 29.06 and 29.25 ppm, 31.75 ppm, and 32.61 ppm. However, these small differences are not signals exclusive to an ester. A pronounced peak appears at 180 ppm (data not shown), but it corresponds to oleic acid. In conclusion, the spectra are very similar and do not allow for the identification of the ester.

**Figure S6.** 13C-NMR of the samples before (Figure S6A) and after the reaction (Figure S6B).
